# Potential Therapeutic Targets of Resveratrol, a Plant Polyphenol, and Its Role in the Therapy of Various Types of Cancer

**DOI:** 10.3390/molecules27092665

**Published:** 2022-04-21

**Authors:** Saleh A. Almatroodi, Mohammed A. Alsahli, Abdullah S. M. Aljohani, Fahad A. Alhumaydhi, Ali Yousif Babiker, Amjad Ali Khan, Arshad Husain Rahmani

**Affiliations:** 1 Department of Medical Laboratories, College of Applied Medical Sciences, Qassim University, Buraydah 51452, Saudi Arabia; smtrody@qu.edu.sa (S.A.A.); shly@qu.edu.sa (M.A.A.); f.alhumaydhi@qu.edu.sa (F.A.A.); ababkr@qu.edu.sa (A.Y.B.); 2 Department of Veterinary Medicine, College of Agriculture and Veterinary Medicine, Qassim University, Buraydah 51452, Saudi Arabia; jhny@qu.edu.sa; 3 Department of Basic Health Sciences, College of Applied Medical Sciences, Qassim University, Buraydah 51452, Saudi Arabia; akhan@qu.edu.sa

**Keywords:** resveratrol, cell signaling pathways, cancers, clinical trials, synergistic effect

## Abstract

Cancer is among the most prominent causes of mortality worldwide. Different cancer therapy modes employed, including chemotherapy and radiotherapy, have been reported to be significant in cancer management, but the side effects associated with these treatment strategies are still a health problem. Therefore, alternative anticancer drugs based on medicinal plants or their active compounds have been generating attention because of their less serious side effects. Medicinal plants are an excellent source of phytochemicals that have been recognized to have health-prompting effects through modulating cell signaling pathways. Resveratrol is a well-known polyphenolic molecule with antioxidant, anti-inflammatory, and health-prompting effects among which its anticancer role has been best defined. Additionally, this polyphenol has confirmed its role in cancer management because it activates tumor suppressor genes, suppresses cell proliferation, induces apoptosis, inhibits angiogenesis, and modulates several other cell signaling molecules. The anticancer potential of resveratrol is recognized in numerous in vivo and in vitro studies. Previous experimental data suggested that resveratrol may be valuable in cancer management or improve the efficacy of drugs when given with anticancer drugs. This review emphasizes the potential role of resveratrol as an anticancer drug by modulating numerous cells signaling pathways in different types of cancer.

## 1. Introduction

Cancer is a leading public health problem worldwide in terms of morbidity as well as mortality. Chemotherapy, radiation, and surgical removal of tumors are some of the most common cancer treatment options, but these traditional treatments carry several serious side effects. Paresthesia, vomiting and nausea, fatigue, chronic pain, and anorexia are among symptoms of therapy-related side effects in cancer patients [1,2]. Therefore, health sciences researchers have focused mainly on medicinal plants or natural compound with anticancer properties to overcome the complications of currently used drugs.

Clinical trial-based studies have demonstrated the beneficial effects of herbal medications on immunological regulation, survival, and quality of life in cancer patients, whether used alone or in conjunction with conventional therapies [3]. As a result, using a combination of herbal medications with diverse mechanisms may enhance the therapeutic efficacy of the treatments synergistically [4]. In this regard, resveratrol has been reported to play an important role in disease management by altering a variety of biological processes, including cell-signaling pathways.

**Figure 1:**
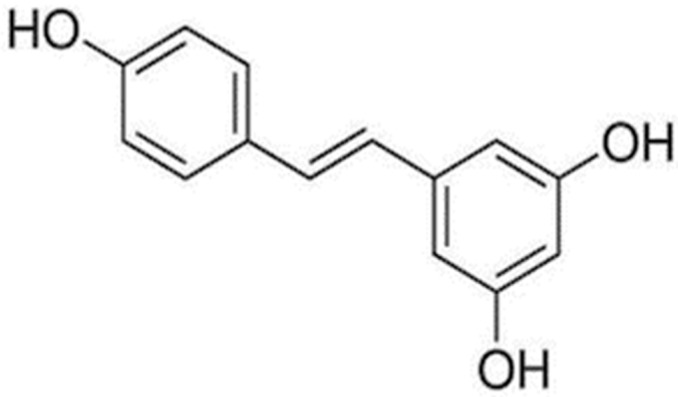
Chemical structure of resveratrol.

Resveratrol is a well-known polyphenolic molecule (Figure 1) and has been found in a range of foods, including vegetables, fruits, and chocolate. Moreover, it is a well-known component of grapes and wines that contain it in modest concentration [5,6]. It is a member of the stilbene family [7], with two phenol rings that are joined by an ethylene bridge. On the basis of chemical structure, there are two isomeric forms of resveratrol, including cis- and trans-resveratrol [8].

Resveratrol has been shown to possess a variety of health-promoting effects, including anti-inflammatory, hepato-protective, neuroprotective, and oxidant activities. In addition, resveratrol is known to protect hepatic cells from oxidative stress by increasing the activity of an antioxidant enzyme and altering the gene expressions [9]. It is also reported to affect various endogenous antioxidant enzymes, affecting their expression and activity [10,11,12], and improve urinary protein excretion, renal oxidative stress, and renal dysfunction [13]. More significantly, resveratrol has been revealed to sensitize numerous resistant cancer cells to anticancer drugs through overcoming chemoresistance mechanisms [14] and promoting the efficacy of anticancer drugs at a low dosage [15]. It has been reported that resveratrol has cytotoxic effects against numerous tumor cells, such as lymphoid, myeloid, breast, cervix, skin, ovary, prostate, stomach, colon, liver, and thyroid carcinoma cells [16,17,18]. Moreover, medicinal plants or their bioactive compounds show a role in cancer prevention through modulating various cell signaling pathways [19,20,21].

This review thus aims at providing an overview of the role of resveratrol in different types of cancer, including mechanisms of actions and vital role in modulating cell-signaling pathways. Moreover, the bioavailability and synergistic effect of resveratrol in combination with anticancer drugs are discussed in detailed.

## 2. Mechanisms of Action of Resveratrol in Cancer Managements

**Figure 2:**
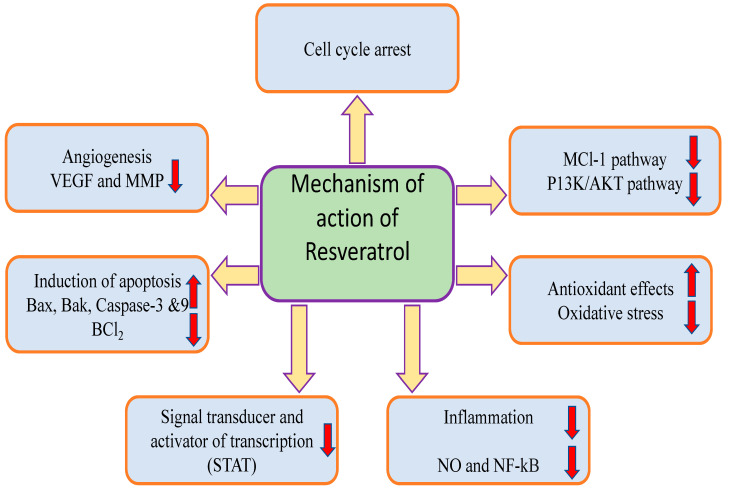
Mechanism of action of resveratrol in cancer management through modulating cell signaling pathways.

Resveratrol plays a significant role in the inhibition of cancer development and progression through the modulation of various cell signaling pathways as described below (Figure 2). 

### 2.1. Antioxidant Effect

The antioxidant activity of resveratrol is associated with its ability to remove reactive oxygen species [22], and as such, its antioxidant potential plays a role in the inhibition of various types of pathogenesis. Resveratrol acts as a potent antioxidant through the Nrf2/HO-1 signaling pathway, increasing glutathione peroxidase (GPx), superoxide dismutase (SOD), and catalase (CAT) activities and HO-1 protein levels, as well as decreasing lipid peroxidation [23]. Treatment with a low concentration of resveratrol significantly increased SOD activity in prostate cancer, liver cancer, and breast cancer cells, and resveratrol-induced SOD2 expression was observed in cancer cells [24]. As a chemo-preventive agent, resveratrol chronic supplementation decreased DNA leukocytic damage, changing the prooxidant/antioxidant balance in a rat model of colon carcinogenesis [25].

### 2.2. Inflammation

Inflammation is one of the major factors that promotes the progression of tumors, increases cancer risk, and supports metastatic spread [26,27]. Moreover, during tumor development, inflammatory mediators, including reactive oxygen species, cytokines, and reactive nitrogen species derived from tumor-infiltrating immune cells, cause the induction of epigenetic changes in silence tumor suppressor genes and pre-malignant cells [28].

Anti-inflammatory drugs are available in a variety of forms; however, some of them have negative side effects. The analgesic effects of resveratrol showed that it reduced the number of writhes and improved the time and threshold for pain in mice. According to the anti-inflammatory findings, resveratrol has the potential to lower ear oedema, inhibit NO generation, suppress white blood cell and pleurisy exudates, and increase SOD activity in serum [29]. Resveratrol considerably inhibited the effect of lysophosphatidylcholine on enzyme activity, the expression of TLR-4, NF-κBp65, and the secretion of proinflammatory cytokine. Furthermore, resveratrol and TLR-4 shRNA transfection suppressed lysophosphatidylcholine-induced injury and inflammation through inhibiting the TLR-4/MyD88/NF-κB signaling pathway. On the other hand, transfection with a Toll-like receptor -4 cDNA enhanced LPC-induced injury and inflammation repealed the protective effects of resveratrol [30].

### 2.3. Tumor Suppressor Gene

Resveratrol significantly reduced the growth of rectal cancer cells and induced apoptosis. It has been reported that the expression of proteins such as PTEN (Phosphatase and tensin homolog), caspase-3, and p53 increases after resveratrol administration [31]. Furthermore, the findings have suggested that resveratrol might be employed as an antiproliferative and apoptosis-inducing drug due to its ability to upregulate PTEN [31]. In addition, resveratrol inhibits the MTA1/HDAC complex, which inhibits the Akt pathway, and hence causes acetylation and reactivation of PTEN [32]. Moreover, the MTA1/HDAC unit is one of the negative regulators of PTEN that encourages prostate cancer development and survival pathways that is suppressed by resveratrol’s MTA1 inhibitory action [32]. The binding of resveratrol to integrin alphaVbeta3, particularly the beta3 monomer, is needed for the stilbene signal to be translated into p53-dependent apoptosis [33]. Resveratrol overwhelms tumor promoter-induced cell transformation, the expression of p53 protein and the transactivation of p53 activity in the same cell line as well as at the same dosage. Moreover, resveratrol-induced apoptosis only happens in cells expressing wild-type p53 (p53+/+), whereas it is not observed in p53-deficient (p53−/−) cells [34]. Another study discovered that ERKs and p38 kinase formed a complex with p53 when treated in the presence of resveratrol, resulting in resveratrol-induced p53 activation mediated by ERKs and p38 kinase and apoptosis by phosphorylating p53 at serine 15 [35].

### 2.4. Apoptosis

Resveratrol has an important function in the triggering of apoptosis, which inhibits the growth and development of cancer cells. With the downregulation of H-Ras, it significantly reduces Akt activity, which is critical for accelerating Bax translocation to mitochondria. Furthermore, resveratrol increases the amount of apoptotic cell death in leukemic cells by inhibiting Akt activity [36]. Resveratrol causes an increase in Bax expression and a reduction in bcl2 expression that are time and dosage dependent. Furthermore, combining resveratrol and prednisolone therapy resulted in a synergistic impact on Bax overexpression and Bcl_2_ downregulation [37]. Previous research has connected the resveratrol-induced growth inhibition with apoptotic cell death in cancer cell lines as well as arrest of the cell cycle [38,39]. A cellular abundance of the oncogene suppressor protein p53, serine phosphorylation of p53, and plenty of c-jun, c-fos, and p21 mRNAs were also increased by resveratrol. Moreover, inhibition of the MAPK pathway through either H-ras antisense transfection or PD 98059 obstructed these resveratrol-induced effects. Addition of pifithrin-alpha or transfection of p53 antisense oligonucleotides caused decreased resveratrol-induced p53 and p21 expression. Moreover, resveratrol induced apoptosis in both used cancer cell lines [40]. Resveratrol induced a breakdown of the mitochondrial transmembrane potential in leukemia cells and swelling of isolated rat mitochondria. Moreover, breakdown of transmembrane potential was not caused by the activation of caspase-8 or Bid, as no significant cleavage of these proteins could be detected in the induction phase of resveratrol-induced apoptosis. Inhibition of mitochondria membrane potential breakdown and of reactive oxygen species generation by N-acetylcysteine or by overexpression of Bcl-2 protein stopped apoptotic induction by resveratrol [41] and Bax mitochondrial translocation [42]. Resveratrol induces apoptosis primarily through a Bak- but not a Bax-mediated AIF-dependent mitochondrial apoptotic signaling cascade [43] in which Bim may provide the power to activate Bak, thus contributing subsequent apoptosis [43].

### 2.5. Angiogenesis

Angiogenesis, or the formation of new blood vessels, is important for cancer development and progression, and it is linked to an invasive phenotype and a poor prognosis [44,45]. Regulating the angiogenesis process, on the other hand, is a key step in cancer prevention and suppression. The administration of resveratrol decreased the expression of vascular endothelial growth factor (VEGF) in glioma cells and the proliferation of human umbilical vein endothelial cells [46]. Resveratrol at doses of 2.5 and 10 mg/kg considerably reduced the tumor weight, tumor volume, and metastasis to the lung in mice bearing highly metastatic Lewis lung carcinoma tumors. In addition, resveratrol inhibited DNA synthesis most strongly in cancer cells, and resveratrol at 100 micromol/L increased apoptosis and decreased the S-phase population. Resveratrol inhibited tumor-induced neovascularization in an in vivo model. Furthermore, resveratrol meaningfully inhibited the formation of capillary-like tube formation from human umbilical vein endothelial cells at concentrations of 10–100 micromol/L [47].

According to a pioneering research study, resveratrol suppresses human ovarian cancer growth and angiogenesis by reducing HIF-1 and VEGF production, suggesting a new putative mechanism for the anticancer effect of resveratrol [48]. In addition, resveratrol has been shown to have an angiogenic impact throughout the progression of hepatocellular carcinogenesis, perhaps by blocking the production of VEGF via HIF-1 downregulation [49]. Furthermore, resveratrol has been reported to suppress VEGF expression and significantly reduce cell proliferation, suggesting that it may have an osteosarcoma effect by lowering the production of VEGF in tumor cells [50].

Another indication of resveratrol’s angiogenic impact is that cotreatment with resveratrol and 5 FU reduced tumor development as compared to the control group, and this growth inhibitory effect was connected to alterations in VEGF expression levels. Furthermore, when resveratrol and 5 FU were given together, the number of microvascular vessels was reduced compared to the control group [51].

### 2.6. PI3K/Akt Pathway

Over-activation of PI3K/Akt is typically caused by changes in the growth factor receptor expression [52], and PI3K has been linked to tumor invasion, development, and angiogenesis [53]. Inhibition of the PI3/Akt pathway, on the other hand, is critical for prevention and treatment of cancer. Autophagy is induced by resveratrol, which decreases the production of the AKT/mechanistic target of the rapamycin signaling pathway-associated proteins [54]. Furthermore, resveratrol protects IPEC-J2 cells from oxidative stress via the PI3K/Akt-mediated Nrf2 signaling pathway, suggesting that resveratrol might be very useful against intestinal damage [55]. The effects of resveratrol and its causal mechanism on hepatocellular carcinoma were investigated. It was established that resveratrol inhibited the proliferation and viability of HCC cells and resveratrol-induced cell apoptosis. In addition to the inhibition of cancer cell migration, resveratrol enhanced the phosphorylation of DLC1 by AKT. These findings demonstrate that resveratrol inhibits the proliferation and migration via a SIRT1-initiated post-translational modification of the PI3K/AKT pathway [56]. Another study reported that resveratrol may be a promising candidate in colon cancer treatment, and the anticancer activity may be arbitrated through inactivating PI3K/Akt signaling via upregulating BMP7 to at least decrease the phosphorylation of PTEN [57].

### 2.7. Signal Transducer and Activator of Transcription 3

Resveratrol effectively inhibits cell proliferation, triggers apoptosis, and is able to inactivate STAT3 signaling. Furthermore, resveratrol has been shown to promote autophagic and apoptotic activity in ovarian cancer cells, presumably by inactivating STAT3 signaling [58]. The effect of resveratrol on osteosarcoma stem cells and the possible molecular mechanisms were examined. It was reported that resveratrol inhibited cell viability, tumorigenesis, and self-renewal ability of cancer cells, while showing no significant inhibitory effects on normal osteoblast cells [59]. Moreover, resveratrol treatment inhibited JAK2/STAT3 signaling and decreased cytokine synthesis [59]. Furthermore, resveratrol reduced hypoxia-induced phosphorylation of STAT3 and decreased p-STAT3 levels in human glioma cells [60].

Human breast cancer cells treated with cancer-associated fibroblasts (CAFs)-conditioned media were inhibited in their proliferation, migration, and invasion by resveratrol. Resveratrol also suppressed Sox2 expression and activated Akt and STAT3 that are induced by cancer-associated fibroblasts [61]. Resveratrol blocks the STAT3 signaling pathway via induction of *SOCS-1*, thus attenuating STAT3 phosphorylation and proliferation in squamous cell carcinoma of the head and neck cells [62]. Resveratrol shows a potent proliferative effect in part via the suppression of STAT3 phosphorylation and Mcl-1 and cIAP-2 expression in HTLV-1-infected T cells [63]. Furthermore, resveratrol has a potent proliferative impact on MT-2 and HUT-102 cell lines, which is mediated in part by the inhibition of STAT3 phosphorylation [63].

### 2.8. Cell Cycle

The effect of resveratrol in enhancing the efficacy of docetaxel in the treatment of prostate cancer was examined. The cancer cell lines were treated with resveratrol, docetaxel, and a combination, and apoptosis/cell cycle progression was evaluated. Moreover, in C4-2B cancer cells, the combination upregulated the *p53* expression and cell cycle inhibitors, which, in turn, inhibited the expression of CDK4, cyclin D1, and cyclin E1 and encouraged Rb hypo-phosphorylation, thereby blocking the transition of cells in the G_0_/G_1_ to the S phase. Moreover, the suppression of cyclin B1 and CDK1 expression from both used cell lines inhibited the progression of cells in the G_2_/M phase [64].

Resveratrol induced noticeable growth inhibition in five of the cell lines. After treatment with resveratrol, most of the cell lines were arrested in the S phase of the cell cycle, and induction of apoptosis was seen. In addition, only a higher dose of resveratrol (300 microM) seemed to decrease cyclin D1 mRNA and Seg-1 cells expressed basal levels of cox-2, which was additional induced by resveratrol [39]. Cell cycle analysis showed that that resveratrol may induce cell cycle arrest in the G0/G1 phase via downregulating the expression levels of cyclin D1, cyclin-dependent kinase (CDK)4 and CDK6, and upregulating the expression levels of the p21 and p27 and CDK inhibitors [65].

Resveratrol treatment was further found to strongly arrest the progression of cell cycle in the G_0_/G_1_, significantly inhibited proliferation of cells, and encouraged apoptosis in a concentration and time dependent manner [66] and other study also reported that resveratrol induces cell cycle arrest [67].

## 3. Resveratrol: Role in Various Cancer Therapy

Cancer is the most often diagnosed disease and is among the top causes of mortality in the world. The existing therapeutic strategies of cancer treatment are costly, and they also have a variety of side effects. Therefore, there is a strong need to develop more acceptable, effective, and less problematic sorts of therapy modules for the prevention and management of cancer.

**Figure 3:**
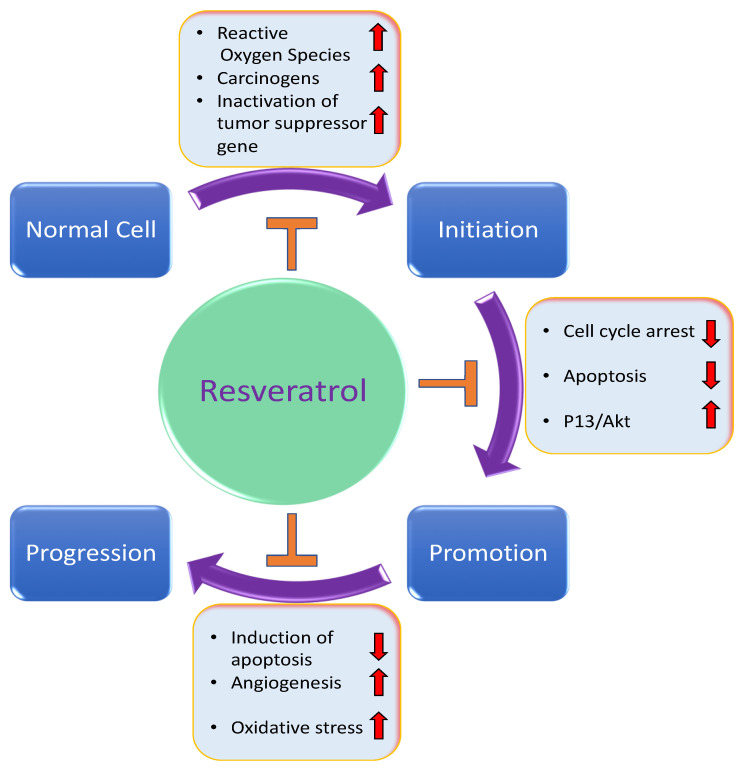
Resveratrol role in the cancer management through inhibition of initiation, promotion, and progression of carcinogenesis and modulate of key cell signaling molecules.

According to various investigations, resveratrol has an important role in cancer treatment and management because of its potential to inhibit the initiation, progression, and promotion of carcinogenesis, as well as in the modulation of critical cell signaling molecules (Figure 3).

The specific role of resveratrol in management of various types of cancer are presented as below (Table 1).

### 3.1. Head and Neck/Oral Cancer

Head and neck cancer is the leading cause of cancer-related mortality worldwide. Resveratrol has been shown to play an important role in the prevention of head and neck cancer growth and development in laboratory trials. Moreover, a xenograft nude mice model was used to investigate the impact of resveratrol in the progression of head and neck squamous cell carcinoma. According to the findings, resveratrol was found to improve the mRNA level of regenerating gene III (REG III) in vivo [68]. Moreover, some in vivo studies suggested that resveratrol rendered head and neck squamous cell cancer more sensitive to irradiation and cisplatin [68]. Resveratrol is shown to inhibit oral squamous carcinoma cell SCC-25 growth and DNA synthesis significantly in a concentration-dependent manner. Furthermore, a combination of resveratrol and quercetin resulted in a gradual but significant increase in quercetin’s inhibitory impact on cell proliferation and DNA synthesis [69]. Another study was conducted to look into the mechanisms involving resveratrol to induce apoptosis. Resveratrol administration reduced the cell viability in a time- and dose-dependent manner while increasing the apoptotic cell ratio in oral squamous cell carcinoma cells, including CAL-27, SCC15, and SCC25 cells [70]. Furthermore, after treatment with resveratrol, oral squamous cell carcinoma cell CAL-27 cells displayed numerous lines of apoptotic exhibition and decreased cell migration, invasion, and epithelial-mesenchymal transition-inducing transcription factor. Overall, the results established the inhibitory effect of resveratrol in human oral squamous cell carcinoma cell cells through the mitochondrial pathway and showed that resveratrol is capable of inhibiting cell invasion as well as migration by inhibiting the epithelial-mesenchymal transition -inducing transcription factors [70].

The effects of resveratrol on oral squamous cell carcinoma cell migration, adhesion, and invasion were investigated in recent research. The MTT assay result showed that the adhesion of an oral squamous cell carcinoma cell line KB cell treated with 100 μmol of resveratrol for 1 or 2 h was decreased by 49.92 and 58.21 %, respectively. Moreover, in the Transwell assay, the migratory and invasive abilities of cancer cells treated with 100 μmol resveratrol were decreased by 43.98 and 37.69 %, correspondingly [71]. Resveratrol inhibited proapoptosis linked signals and autophagy and increased the phosphorylation of AMPK. Resveratrol also increased autophagic mRNA gene expression, including Atg5, Atg12, Beclin-1, and LC3-II in human oral cancer CAR cells [72].

Inhibition of proliferation of tested cell lines was noted after treatment with resveratrol in a concentration- and time-dependent fashion. Investigation of the cell cycle analysis presented that treatment with resveratrol induced cell cycle arrest in the G2/M phase and increased the expression of cyclin A2, and cyclin B1 and phospho-cdc2 (Tyr 15) in the cancer cells. It caused a noticeable increase in the percentage of apoptotic cells. Resveratrol displayed an inhibitory effect on the proliferation of OSCC oral cancer cells via the induction of apoptosis and G2/M phase cell cycle arrest [73].

### 3.2. Esophageal Cancer

In rat models, the effects of resveratrol on the progression of reflux esophagitis from metaplasia to dysplasia to esophageal adenocarcinoma were studied. The resveratrol-treated group was found to have stable morphological appearances, less esophagitis, and lower occurrences of metaplasia and esophageal cancer. Moreover, the carcinogenic effects and progression to metaplasia were both reduced by this stilbenoid [74]. Moreover, this phytoalexin suppresses esophageal squamous cell carcinoma cell growth by inducing cell cycle arrest during the sub-G_1_ phase in a dose-dependent manner. Furthermore, resveratrol-induced autophagy in esophageal squamous cell carcinoma is independent of the AMPK/mTOR pathway [75].

The potential mechanisms of resveratrol in the inhibition of esophageal tumorigenesis were examined. It was reported that a N-nitrosomethylbenzylamine treatment group animal showed high expression of COX-1 mRNA in tumor tissues. The higher expression of COX-1, the increased levels of PGE(2) synthesis, and the upregulated COX-2 expression were significantly decreased by administering resveratrol. Finally, the study showed that resveratrol suppressed NMBA-induced esophageal tumorigenesis through targeting cyclooxygenase and prostaglandin E(2). Consequently, it may be an encouraging natural anticarcinogenesis agent in the prevention and treatment of human esophageal cancer [76].

A study was conducted to investigate resveratrol-induced apoptosis in esophageal cancer cells, as well as the link between apoptosis and Bcl-2 and Bax expression. The findings revealed that resveratrol can promote apoptosis in esophageal cancer cells. Downregulating the expression of apoptosis-regulated genes such as Bcl-2 and upregulating the expression of apoptosis-regulated genes such as Bax can be involved in the prevention of this kind of apoptosis [77].

### 3.3. Lung Cancer

Resveratrol activates the tumor suppressor gene p53 as well as its proapoptotic modulator PUMA. Furthermore, p53-independent apoptosis has been shown to reduce the expression of phosphorylated Akt-mediated NF-B, as indicated by the downregulation of antiapoptotic proteins Bcl-2 and Bcl-xl in lung cancer. Apoptosis was also induced in TRAIL-resistant lung cancer cells when resveratrol was added in the therapeutic strategy [78].

Resveratrol had an antitumor effect via inhibition of cell proliferation and encouragement of cell apoptosis in cancer cells dose dependently. Moreover, resveratrol caused an increase in the relative expression of Beclin1 and LC3 II/I, whereas p62 expression decreased, suggesting that resveratrol induced autophagy in lung cancer cells. Furthermore, resveratrol increased expression of SIRT1 as well as SIRT1 activator SRT1720-induced autophagy of lung cancer cells. SIRT1 knockdown decrease resveratrol-induced autophagy considerably. These finding designated that resveratrol might prompt autophagy via SIRT1 expression upregulation [79].

The effects of resveratrol on small-cell lung cancer cell proliferation and apoptosis were evaluated [80]. According to the findings of this study, resveratrol causes time-dependent cell viability reduction. Furthermore, resveratrol-treated cells were reported to have higher apoptotic rates, which were connected to mitochondrial depolarization and cytochrome c release from mitochondria. Furthermore, resveratrol promoted cisplatin-induced cancer cell inhibition, as seen by lower viability and increased apoptosis [80]. In another investigation, lung cancer cells were shown to be more susceptible to resveratrol therapy than human bronchial epithelial Beas-2B cells. Furthermore, this natural product inhibits cell viability and cell proliferation and induces substantial apoptosis in lung cancer cells [146].

Curcumin and resveratrol were tested for their combined chemopreventive efficacy in benzopyrene-induced lung carcinogenesis. Curcumin and resveratrol supplementation of benzopyrene-treated rats significantly reduced lipid peroxidation, glutathione levels, and enzyme activity of drug-metabolizing enzymes. A therapy having a combination of curcumin and resveratrol significantly increased the activities of glutathione reductase, superoxide dismutase, and Glutathione-S-transferase in benzopyrene-treated rats. Furthermore, this combination therapy improved the lung tissue architecture significantly [147]. Exposure of human non-small-cell lung cancer such as A549, H460, H1975, and PC-9 cells to minimal concentrations of resveratrol and erlotinib synergistically decreased cell viability and induced cell apoptosis. Additionally, resveratrol synergistically enhanced erlotinib-induced apoptosis was involved in reactive oxygen species production. Furthermore, cotreatment with resveratrol and erlotinib repressed the expressions of antiapoptotic proteins, including Mcl-1 and surviving, while promoting PUMA and p53 expression and caspase-3 activity. Consequently, a small interfering RNA reduction of PUMA and a high expression of survivin considerably attenuated lung cancer cells apoptosis induced by the combination of resveratrol and erlotinib drugs [81].

### 3.4. Gastric Cancer

Plasma interleukin-6 (IL-6) was shown to be increased in gastric cancer patients [82]. Furthermore, resveratrol reduced the activation of matrix metalloproteinases IL-6-induced cancer cell invasion in a gastric cancer cell line model. Furthermore, result showed that IL-6-induced cancer cell invasion is dependent on the activation of the Raf/MAPK pathway and that resveratrol may suppress this pathway activation [82]. Resveratrol reduced the expression of Wnt signaling pathway components, cyclin D1 and c-myc at the mRNA, and protein levels in gastric cancer cells, causing apoptotic morphological alterations [83]. In general, resveratrol stops gastric cancer cells from growing by inhibiting the Wnt signaling pathway [83]. This stilbenoid helped SGC7901/Doxorubicin cells regain doxorubicin sensitivity, reduced aggressive biological characteristics, promoted cell death in vitro, and stopped tumor development in vivo. Furthermore, SGC7901/Doxorubicin cells undergo an epithelial-mesenchymal transition that is induced by Akt activation as well as PTEN activation. Resveratrol inhibits the Akt pathway, which leads to EMT reversal [148]. An important study was performed to examine the effect of resveratrol on apoptosis in gastric cancer cells as well as its molecular mechanisms of action. The results demonstrated that resveratrol was capable of intentionally inhibiting the viability of cancer cells in a dose- and time-dependent fashion. In the presence of resveratrol, the proportion of apoptotic cells was increased in a dose-dependent fashion. Moreover, resveratrol induced S-phase arrest of cancer cells. Moreover, the levels of the cleaved caspase-3, proapoptotic proteins Bax and cleaved caspase-8 were upregulated in a dose-dependent way, while the antiapoptotic protein Bcl-2 was downregulated dose dependently. Importantly, resveratrol was able to inhibit viability and induce apoptosis in cancer cells through decreasing NF-κB activation [84]. Apoptosis was induced in human gastric carcinoma cells, and cleaved PARP and caspase-3 was activated by resveratrol. Following this, the mitochondrial membrane potential of cells dissipated after the cells were treated with resveratrol. Furthermore, it was observed that downregulation of procaspase 9 and cytochrome c released from mitochondrial to the cytosol and the ratio of Bax/Bcl-2 expression was enhanced in the treated cells. Resveratrol promotes apoptosis in cancer cells and stimulates apoptosis via the mitochondrial route [149]. In human gastric cancer cells, the resveratrol role as anticancer has been described via different mechanism treatment with resveratrol concentration considerably inducing apoptosis and DNA damage. This was due to the increased generation of reactive oxygen species following resveratrol treatment because incubation of cells with SOD catalase reduced the resveratrol-induced cellular apoptosis [150]. Resveratrol-induced suppression of human gastric adenocarcinoma cell proliferation may be partially reliant on NO production, and it has been proposed that resveratrol inhibits cell proliferation by interfering with the effect of endogenously generated ROS [85].

### 3.5. Gall Bladder Cancer

TG2 inhibition might reduce resveratrol’s cytotoxic impact on cholangiocarcinoma and gallbladder cancer. It was reported that cell growth was significantly slowed after treatment with resveratrol. The inhibitors effectively lowered TG2 activity without affecting protein content [151]. The role of resveratrol in the proliferation and apoptosis of gallbladder carcinoma cell line and fibroblast 3T3 cell line investigated. It was reported that resveratrol clearly suppressed the proliferation in concentration and time dependent fashion and induced apoptosis of tumor cells. The maximum apoptosis rate of tumor was 30. 52%, in the treatment group as compared with the control group whose Gl cells rose from 34. 88% to 55.47% and S cells decreased 8.41–17.54%, which presented a clear occurrence of G_0_/G_1_ blocking. The findings showed that this substance inhibits tumor cell growth in a dose- and time-dependent manner and that resveratrol may promote tumor cell death [86].

### 3.6. Bile Duct Cancer

Cholangiocarcinoma cells secrete IL-6 and a little quantity of IL-8 in response to fibroblast-stimulated secretion in the conditioned media of cholangiocarcinoma-derived malignancy. Although IL-6 was shown to increase cell migration in cholangiocarcinoma cells, resveratrol was found to effectively counteract this effect [87]. More importantly, resveratrol has been shown to stop cancer-associated fibroblasts from secreting IL-6. While the conditioned medium from cancer-associated fibroblasts dramatically increased IL-6-mediated cholangiocarcinoma cell motility, the conditioned medium from cancer associated fibroblasts pretreated with resveratrol entirely prevented cancer cell movement [87].

### 3.7. Liver Cancer

The anticancer effects of resveratrol on paclitaxel in liver cancer cells were investigated in a recent in vitro study. In cancer cells, PA-L and PA-H displayed growth inhibitory effects, whereas resveratrol increased these growth inhibitory effects [152]. Furthermore, compared to other treatment groups, the liver cancer cells displayed the highest apoptosis after a treatment using a combination of resveratrol plus PA-H, and after additional treatment of resveratrol, both the apoptotic cells of two PA concentrations were found to be elevated [152]. Resveratrol inhibited proliferation, viability, invasion, and migration of liver cancer cells pointedly in a time- and dose-dependent way, demonstrating that resveratrol employed antitumor effects in liver cancer. Additionally, the relative expression of autophagy-related proteins Beclin1 and LC3 II/I ratio was increased, whereas the expression of p62 was decreased by treatment with resveratrol. The LC3+ puncta formation, which characterized autophagosome formation, was also evidently dose dependently enhanced through resveratrol treatment, suggesting that resveratrol induced autophagy in liver cancer cells. Furthermore, treatment with autophagy inhibitor 3-methyladenine countered the inhibitory effect of resveratrol on liver cancer cell invasion, migration, and proliferation, demonstrating that suppressing autophagy may hinder the antitumor effect of resveratrol [88].

The effects of resveratrol on hepatocellular carcinoma and the mechanisms behind them were studied. The results showed that resveratrol reduced the viability and growth of hepatocellular cancer cells. Furthermore, resveratrol was shown to cause cell death by enhancing hepatocellular carcinoma apoptosis. Furthermore, resveratrol therapy suppressed the migration of cancer cells by inhibiting the PI3K/Akt pathway [56]. Resveratrol treatment showed a role in the downregulation of p38 MAP kinase and cyclin D1, Akt and Pak1 expression, and activity in liver cancer cells, signifying that the growth inhibitory activity of resveratrol is linked with the cell proliferation and survival pathways downregulation. In addition, resveratrol-treated cells showed enhanced ERK activity, signifying possible sensitization to apoptosis [153].

Resveratrol inhibits the HGF-c-Met signaling pathway, which has a potent anticancer impact on hepatocellular carcinoma cells. Furthermore, resveratrol reduced hepatocellular carcinoma cells’ anchorage-dependent and independent proliferation in a dose-dependent manner [89]. Moreover, resveratrol’s anticancer efficacy was validated in a xenograft model that showed its significant inhibitory effect on tumor development in vivo [89]. Hepatic stellate cells are reported to promote angiogenesis in hepatocellular carcinoma via increasing Gli-1 expression that induces the formation of ROS and increases the invasiveness of hepatocellular carcinoma cells. Resveratrol has been reported to inhibit angiogenesis and to reduce ROS formation in hepatic stellate cells [154]. Resveratrol has been found to enhance the intracellular content of ceramide and to increase the expression of enzymes involved in the de novo ceramide production pathway whether used alone or in combination with palmitic acid. Furthermore, in lipid overload situations, resveratrol significantly decreased intracellular triacylglycerol buildup. Overall, some evidence suggests that resveratrol has a protective impact on liver cells in a lipid excess condition, at least in part [90].

### 3.8. Pancreatic Cancer

Resveratrol decreases the expression of nutrient-deprivation autophagy factor-1 in pancreatic cancer cells by inducing cellular ROS accumulation and activating Nrf2 signaling. More notably, the targeting of nutrient-deprivation autophagy factor-1 by means of resveratrol can increase the sensitivity of pancreatic cancer cells to gemcitabine [155]. A study was aimed to assess the chemopreventive effects of resveratrol and apocynin in pancreatic carcinogenesis. Resveratrol- and apocynin-treated hamsters exhibited an important decrease in the incidence of pancreatic cancer. Moreover, resveratrol and apocynin inhibited cell proliferation of human and hamster pancreatic cancer cells through preventing the G_1_ phase of the cell cycle [91].

Pancreatic cancer cells were inhibited from proliferating and undergoing apoptosis due to resveratrol treatment. The activation of an AMP-activation protein kinase might be responsible for such physiologic consequences [156]. According to a research study, resveratrol can lead to a decrease in cell proliferation in a dose- and time-dependent manner. Furthermore, resveratrol was found to have a dose-dependent effect in the activation of apoptosis in pancreatic cancer cells [92]. A study was conducted to see if resveratrol’s anticancer properties were connected to VEGF-B. According to these findings, this stilbenoid therapy was found to cause apoptosis. Additionally, it reduced tumor development and elevated Bax expression in capan-2 cells. Furthermore, resveratrol therapy increased the levels of VEGF-B mRNA and protein [93]. The effect of a macrophage inhibitory cytokine in the growth inhibition of pancreatic cancer cell lines caused by resveratrol was investigated. The findings revealed that resveratrol treatment increased the expression of macrophage inhibitory cytokine genes in human pancreatic cancer cells [157]. Cytotoxic and biochemical effects of a newly synthesized polymethoxylated resveratrol analogue in human pancreatic cancer cell lines was investigated. The human pancreatic cancer cell lines BxPC-3 and AsPC-1 were used to test the potential inhibitory effect of the resveratrol derivative on cell proliferation and the underlying mechanisms of this effect. After 7 days of incubation, the resveratrol analogue *N*-hydroxy-*N*′-(3,4,5-trimethoxphenyl)-3,4,5-trimethoxy-benzamidine (KITC) inhibited the growth of BxPC-3 and AsPC-1, arrested cells in the G_0_/G_1_ phase, and depleted cells in the S phase of the cell cycle. Moreover, KITC induced dose-dependent apoptosis in both pancreatic cancer cell lines and was found to meaningfully reduce the in-situ activity of ribonucleotide reductase [94].

### 3.9. Colon Cancer

A recent study based on colorectal cancer reported that at a 50 or 100 μmol/L concentration, resveratrol decreased the cell number as well as increased the percentage of apoptotic or necrotic cells, therefore demonstrating cytotoxicity. Moreover, resveratrol-induced cytotoxicity on HT-29 cells was linked with NADPH oxidase activation and increased levels of histone γH2AX [95].

Another research study looked into the advantages and efficacy of As_2_O_3_ in conjunction with resveratrol for colon cancer therapy. Both As_2_O_3_ and resveratrol can effectively limit cell growth and increase cell death in colon cancer cells according to the findings. The combined impact of both medications on colon cancer cells is more impressive than each drug’s effect alone [96]. Surprisingly, resveratrol’s cytotoxic effects were linked to an increase in oxygen consumption sustained by mitochondrial biogenesis as well as enhanced oxidation of fatty acid. According to the research, resveratrol’s anticancer properties include increasing mitochondrial electron transport chain overload, targeting cancer cell metabolism, and eventually increasing ROS formation [158]. Resveratrol decreased colony expansion and cell proliferation in colon cancer cells but had no effect on normal colon epithelial cells according to the study. Resveratrol inhibited cell proliferation, which was linked to the activation of apoptosis and the G_1_ phase of the cell cycle.

Furthermore, resveratrol therapy reduced the expression of cyclin E2 and the BCL2 apoptotic regulator protein and decreased cyclin D1 expression [97]. The effect of resveratrol on human colon cancer cell lines was investigated through evaluation of cell viability and apoptosis. Result exhibited that exposure of colon cancer cells to resveratrol inhibited cell viability. Moreover, it meaningfully induced apoptosis in both of the used cancer cell lines. The population of apoptotic cells in SW480 and HCA-17 cell lines after resveratrol treatment was 67.2 ± 4% and 59.8 ± 4, respectively. Moreover, cancer cells exposed to resveratrol presented meaningfully lower prostaglandin receptor and cyclooxygenase-2 expression [98]. The antiproliferative as well as proapoptotic effects of resveratrol were evaluated. To assess the toxicity of a relatively low dose of resveratrol, colon cancer cells Caco2 were treated with resveratrol. It was reported that resveratrol reduced the proliferation of colon cancer cells in a time-dependent fashion. Moreover, exposure to 10 µM resveratrol increased the relative proportion of cells in the S phase of the cell cycle from 30.5% to 34.5%, demonstrating a modest cell cycle effect in the S phase. Exposure of colon cancer cells to resveratrol caused in a small increase in the apoptotic population. Though a treatment of cells with 10 µM resveratrol showed no effect on viability, higher doses of resveratrol decreased cell viability [159].

Another research study validated resveratrol’s significance in colon cancer. The data showed that PPARγ was involved in resveratrol-induced apoptosis. It suggested that combining resveratrol with a PPAR agonist might be a promising therapeutic strategy in the treatment of colorectal cancer [99].

### 3.10. Renal Cell Carcinoma

The effect of sitagliptin with or without resveratrol on clear cell renal cancer was investigated in the literature. Sitagliptin and/or resveratrol significantly improved renal function while also significantly increasing tissue antioxidant defenses. Finally, it was hypothesized that the combination of sitagliptin and resveratrol might be a suitable treatment method for improving clear cell renal cancer [100]. Resveratrol has been shown to play a function in renal cell carcinoma, acting as a tumor suppressor in both a dose- and time-dependent way. In addition to the findings of the MTT and cell migration studies, resveratrol was demonstrated to reduce migration and cell viability significantly. Furthermore, resveratrol decreased the expression of the anti-apoptosis gene Bcl 2, while increasing the expression of the pro-apoptosis gene. Resveratrol also inhibited renal cell carcinoma migration and enhanced apoptosis in renal cell carcinomas [101]. In a concentration- and time-dependent way, resveratrol inhibited renal cell carcinoma cell growth, migration, and invasion. Resveratrol-induced inhibition of the Akt and ERK1/2 signaling pathways is thought to have caused these consequences [102]. The potential of combining resveratrol with TRAIL was tested. It was reported that resveratrol can sensitize renal cell carcinoma cells to TRAIL-induced death. Moreover, resveratrol plus TRAIL encourages autophagy and apoptosis in cancer cells. It was shown that the apoptosis is caspase-dependent, and the activation of caspase-3, caspase-8, and caspase-9 was involved in this process. In addition, the expression of XIAP was meaningfully inhibited after resveratrol plus TRAIL treatment in cancer cells. Additionally, a fiber-modified replication-deficient adenovirus, Ad5/35-TRAIL, was produced to test the synergistic effect of resveratrol and TRAIL in vivo [160].

The effect of resveratrol on the activation cascade of STAT3 and STAT5 in renal cell carcinoma cell lines was investigated. According to the findings of this investigation, resveratrol potentially inhibited both constitutive STAT5 and STAT3 activation. Resveratrol also caused S-phase cell-cycle arrest, apoptosis, and suppression of colony formation in renal cell cancer cells. HS-1793 is a resveratrol analogue that may represent a new possibility for overwhelming Bcl-2 resistance conferred by PML protein and mature promyelocytic leukemia nuclear bodies production [103].

### 3.11. Prostate Cancer

It was reported that resveratrol decreased cellular survival and migration and enhanced cell death. Furthermore, it was discovered that resveratrol increased reactive oxygen species concentration and expression of the cell death Bax biomarker while preventing Bcl2 and emphasizing expression of p53. Furthermore, resveratrol significantly increased the expressions of p53 and HIF-1α in prostate cancer cells. Resveratrol promoted cell death and suppressed cell survival, but its effects were overturned after HIF-1α knockdown, signifying that the effects of resveratrol in prostate cancer are mediated through HIF-1α. Finally, results indicated that resveratrol encourages apoptosis through p53/HIF-1α/ROS signaling in prostate cancer cells [161].

Furthermore, the ability of resveratrol to inhibit dihydrotestosterone-induced prostate cancer metastasis has been investigated. Dihydrotestosterone boosted cell viability in prostate cancer cells. However, resveratrol and its combination with bicalutamide dramatically inhibited dihydrotestosterone-induced cell viability as well as apoptotic activity generated by resveratrol therapy. Finally, this research suggested that resveratrol in conjunction with an androgen receptor and CXCR4 antagonists can be utilized to reduce prostate cancer metastatic behavior [104].

The impact of resveratrol on the physiology of prostate-cancer-related fibroblasts in the setting of the tumor microenvironment has also been examined. It was discovered that resveratrol activates the N-terminal mutant transient receptor potential ankyrin 1 channel using a prostate-cancer-related fibroblasts cell line and primary cultures of cancer-associated fibroblasts from prostate tumors [162]. The effect of resveratrol on ARV7 transcriptional activity as well as the possibility of developing resveratrol as a therapy for ARV7-positive prostate cancer were investigated. Furthermore, it has been discovered that resveratrol can reduce the levels of endogenous ARV7 expression [105]. The ability of resveratrol to improve the efficacy of docetaxel therapy in prostate cancer cells has also been studied. The cancer cell lines were given docetaxel, resveratrol, or a combination of the two drugs. It was found that the combination therapy resulted in overexpression of proapoptotic genes. The role of resveratrol-induced apoptosis in prostate cancer cells treated with a known concentration of docetaxel, resveratrol, and resveratrol + docetaxel was examined. The pro- and antiapoptotic protein expressions in cancer cells after treatment with resveratrol, docetaxel, and a combination of both drugs displayed the downregulation of antiapoptotic genes *BCL-2*, *MCL-1,* and *BCL-XL* and the upregulation of proapoptotic genes *BAK, BAX* and, BID. Moreover, upregulation of the apoptotic marker cleaved expression of PARP in the cells treated with the combination of both drugs improved the effectiveness of the resveratrol and docetaxel in inducing apoptosis. Moreover, proapoptotic genes (*BAX, BAK* and BID) were upregulated in which the expression of *BID* and *BAX* was more substantial in the cells treated with both drugs. These outcomes show that resveratrol induces the pathway of apoptosis, therefore helping cell death [64]. In vivo effects of resveratrol treatment on different cancers including prostate cancers was reported [163].

### 3.12. Urinary Bladder Cancer

A combination of rapamycin and resveratrol was thought to be very effective in inhibiting mTOR and PI3K signaling while also inducing cell death in bladder cancer cells. A therapy having both resveratrol and rapamycin was shown to inhibit rapamycin-induced Akt activation. Furthermore, cell death was induced by this combined therapy, and this combination was discovered to be a promising therapeutic option in the treatment of bladder cancer [164]. An investigation to evaluate the metastatic ability of resveratrol to protect from bladder cancer and its possible mode of action was carried out. It was found that resveratrol was able to inhibit the migration, adhesion, and invasion of bladder cancer cells in a concentration-dependent manner. Additionally, resveratrol was found to decrease the expression and secretion of matrix metalloproteinase MMP9 and (MMP)2. Resveratrol also inhibited phosphorylation of cJun N-terminal Kinase and extracellular signal-regulated protein kinase [106]. Resveratrol antitumor activity with different concentrations and its possible mechanisms of action in bladder tumor with different TP53 gene status were evaluated. The results demonstrated that resveratrol decreased cell proliferation and induced DNA damage in all used cell lines and that resveratrol reduced the number of colonies [107]. The effects of resveratrol on bladder cancer cell apoptosis were examined at the molecular level. The findings revealed that resveratrol induced cancer cell cytotoxicity and apoptosis in a dose-dependent manner. Resveratrol inhibited Akt phosphorylation, miR 21, and Bcl 2 protein expression [108]. The effect of resveratrol on human bladder cancer cell oxidative stresses was explored in a dose-dependent manner, and resveratrol was found to induce cell death at high concentrations. Cells pretreated with 2.5 μM were found to be protected from oxidative damage, whereas 50 M μM accelerated cell death and significantly increased the Bad/Bcl-2 ratio. Resveratrol at high doses encouraged cell death of cancer cells, while low doses showed protection. Modulation of the Bcl-2 protein induced through resveratrol might be facilitating this effect [165]. Moreover, resveratrol therapy has been shown to reduce the expression of VEGF and fibroblast growth factor-2, which may contribute to tumor growth inhibition in the xenograft model of bladder cancer [109].

### 3.13. Breast Cancer

A study was carried out to explore the functional significance of resveratrol in breast cancer cell lines in terms of chemosensitivity to adriamycin and to identify the resveratrol-targeted miRNAs and their principal target proteins linked with cell activity. Resveratrol-induced chemosensitivity, cell cycle, and apoptosis were arrested in adriamycin-resistant breast cancer cells after regulation of the critical suppresser, miR-122-5p [110]. Furthermore, the findings suggest that resveratrol may operate as a potential inducer to increase the chemosensitivity of breast cancer cells [110].

Treatments with the combinations of grape seed proanthocyanidins and resveratrol synergistically decreased cell viability as well as posttreatment cell proliferation in both used cell lines. Moreover, treatments with grape seed proanthocyanidins and resveratrol in combination synergistically induced apoptosis in MDA-MB-231 cells via downregulating Bcl-2 expression and upregulating Bax expression [111]. Furthermore, the combined therapy triggered apoptosis by upregulating Bax expression while downregulating Bcl-2 expression. It was reported that a combinational treatment of grape seed proanthocyanidins and resveratrol meaningfully induced apoptosis by 21.8% as compared to 4.1% and 3.4% induced by treatment with resveratrol and grape seed proanthocyanidins alone, respectively. However, the combinational treatment of both drugs directed 24.6% cell death in cancer cells MCF-7, which is better than the 19.1% as caused by grape seed proanthocyanidins and the 5.1% caused by resveratrol combined. The findings suggest that combination treatments kill breast cancer cells synergistically by the induction of apoptosis and by the alteration of DNA methylation [111]. However, resveratrol was shown to inhibit proliferation and colony formation in breast cancer. Furthermore, resveratrol’s antiproliferative actions against breast cancer cells were found to be dependent on EZH2 expression being suppressed via ERK1/2 dephosphorylation [112]. SIRT1 was shown to be involved in the cytotoxic effects of resveratrol on breast cancer cells and on mitochondrial complex I inhibition. The involvement of resveratrol in the decrease of breast cancer cells with stemness markers was recently discovered [166]. Moreover, cotreatment with salinomycin meaningfully potentiated the anticancer effects of resveratrol. Cell cycle arrest, caspase activation, and apoptosis induction in cells treated with a resveratrol-salinomycin combination established the efficiency of the combination [113]. Resveratrol upregulates MICA and MICB through suppressing the c-Myc/miR-17 pathway in breast cancer cells, and it has been shown to increase the cytolysis of breast cancer cells via NK cells [167].

### 3.14. Endometrial Cancer

Administration of resveratrol in endometrial cancer patients has been shown to inhibit cell growth in cancer cells in a concentration-dependent manner. In addition, resveratrol has been reported to improve the abundance of the sub-G_1_ population while also inducing apoptosis. Whether or not resveratrol-mediated autophagy affected the resveratrol antitumor effect in cancer cells by the addition of chloroquine in combination with resveratrol, cell viability was significantly suppressed by a combination treatment of both drugs as compared to resveratrol treatment alone. Furthermore, the combination treatment of both drugs displayed an inclination toward an improved population of double-positive (apoptotic) cells. These data designated that combination treatment with both drugs such as resveratrol and chloroquine may induce better cytotoxicity in cancer cells. Furthermore, annexin V-PI double staining revealed that resveratrol-induced apoptosis was improved through silencing *ATG5* or *7*, while the knockdown of *ATG5* or *7* alone did not affect apoptosis in cells without resveratrol treatment. The findings of the study indicated that resveratrol-induced autophagy may neutralize the anticancer effect of resveratrol in cancer cells [168]. Endometrial cancer cells were given different doses of resveratrol, and their cell cycle regulatory genes, expression of growth signaling genes, and apoptosis-associated genes were investigated [114].

The data indicate that the proliferation of cancer cells was reduced in a concentration-dependent manner by resveratrol administration. Furthermore, gene and protein expression results showed that resveratrol therapy reduced EGF while increasing VEGF in cancer cell lines [114]. Bhat and Pezzuto reported that resveratrol had antiestrogenic and cytostatic activities with human endometrial adenocarcinoma. When human endometrial adenocarcinoma ishikawa cells were treated with resveratrol as a single agent, the estrogen-inducible progesterone receptor was not increased, and progesterone receptor expression induced by treatment with 17beta-estradiol (E(2)) was inhibited by resveratrol in a dose-dependent manner at both the protein and mRNA levels [115]. Moreover, resveratrol initiated suppression of a functional activity of the progesterone receptor as established by the downregulation of alpha (1)-integrin expression induced by E(2) plus progesterone [115].

### 3.15. Cervix Cancer

Resveratrol-induced cell death in cervical cancer cell lines through apoptosis and autophagy has also been studied. In all of the cell lines investigated, resveratrol administration at varied concentrations promoted cell cycle arrest at the G_1_ phase and induced apoptosis. Furthermore, p53 expression in human papillomavirus18 (HPV18) positive cell lines was reduced by resveratrol therapy. However, p53 expression was reported to be increased in human papillomavirus positive16 (HPV16) cell lines by the same therapy [116]. In a recent study, resveratrol was found to have a role in the reduction of matrix metalloproteinase-9 expression and enzymatic activity, as well as the promoter activity of PMA-stimulated metalloproteinase-9. Furthermore, resveratrol was found to decrease metalloproteinase-9 transcription by inhibiting both activator protein 1 transactivation and NF-B. Kim and coworkers reported that resveratrol has the potential to inhibit both nuclear factor B and Activator protein 1 mediated metalloproteinase-9 expression. Thus, resveratrol inhibits the migration and invasion of human metastatic lung and cervical cancer cells [117]. Zhang and colleagues found that resveratrol enhanced the expression of PIAS3 and SOCS3 in cervical cancer cells, which was followed by suppression of the signal transducer and activator of transcription 3 signaling. However, the level of SHP2 in all evaluated cell lines remained unchanged after resveratrol administration [169]. In a pioneering investigation, Hsu et al. found that the cat L-squamous cell carcinoma antigen lysosomal pathway and autophagy are involved in resveratrol-induced cytotoxicity in cervical cancer cells. The data revealed that resveratrol enhances the presence of LC3-II and autophagosomes while also causing GFP-LC3 accumulation. Long-term exposure to this stilbenoid resulted in caspase-3 activation, cytosolic cytochrome c translocation, and apoptotic cell death [118]. Using irradiated HeLa and SiHa cells pretreated with resveratrol prior to ionizing radiation (IR) treatment, clonogenic cell survival experiments revealed increased tumor cell death by IR in a dose-dependent manner. These findings imply that resveratrol affects both cell cycle progression and the cytotoxic response to IR. Resveratrol pretreatment inhibited cell division as indicated by growth curves and triggered an early S-phase cell-cycle checkpoint arrest according to further investigation of cyclooxygenase-1 suppression [119]. An investigation was carried out to explore the cytotoxic effects of styrylquinazoline derivatives (synthetic analogues of resveratrol), and it was shown that cervical cancer growth was most effectively prevented by 8-ADEQ. A G2/M cell cycle arrest was reported while investigating the growth-inhibitory mechanisms of 8-ADEQ. Furthermore, it was shown that 8-ADEQ activates checkpoint kinases 1 and 2 to trigger phosphorylation of the cell division cycle 25C protein [170].

### 3.16. Ovarian Cancer

Interlukin-6 and resveratrol affect in an opposite way the expression of RNA messengers and of microRNAs participating in extracellular matrix remodeling and cell locomotion linked with the invasive properties of ovarian cancer cells. Resveratrol might counteract the Interlukin-6 induction of cell migration in cancer cells via autophagy induction in the cells at the migration front, which was paralleled by downregulation of STAT3 expression and upregulation of ARH-I. Moreover, a Spautin 1-mediated interruption of the BECLIN 1-dependent autophagy revealed the effects of resveratrol while encouraging cell migration. Finally, findings show that resveratrol causes its tumor effect via epigenetic mechanisms, thereby supporting its inclusion in the chemotherapy regimen [171].

Research was conducted to determine the status of ARHI expression and its significance in resveratrol-treated cell growth retardation, signal transduction, and transcription 3 inactivation. ARHI expression is low in ovarian cancer cell lines, but it increased following resveratrol therapy, accompanied by extensive apoptosis, growth arrest, and increased autophagic activity [120]. Analogues of resveratrol inhibited Akt and MAPK signaling and reduced the migration of IL-6 and EGF-treated cells. Finally, resveratrol analogues have been shown to reduce the expression of epithelial mesenchymal transition markers in ascite-derived cancer cells [121].

The effect of resveratrol on the invasiveness of norepinephrine-induced ovarian cancer was investigated. Resveratrol pretreatment was shown to suppress norepinephrine-induced epithelial-to-mesenchymal transition in ovarian cancer cells. Furthermore, resveratrol inhibited NE-induced telomerase reverse transcriptase production by reducing Src phosphorylation and Hypoxia-inducible factor *1-α* (HIF-α). Resveratrol also suppressed norepinephrine-induced slug expression and ovarian cancer invasion [172].

In human ovarian cancer cell lines, the effect as well as the key mechanisms of resveratrol were investigated. The findings show that resveratrol therapy causes apoptotic cell death that is dependent on both dose and time, as well as a transitory increase in ROS formation. Furthermore, resveratrol administration was shown to reduce Notch1 signaling depending on ROS. Resveratrol also increased phosphatase and tensin homolog while decreasing phosphor Akt. The findings showed that resveratrol causes caspase-dependent cell death in ovarian cancer cells by suppressing Notch1 and PTEN/Akt signaling, which is countered by increased ROS production [122].

### 3.17. Uterine Cancer

The involvement of the Wnt signaling pathway in resveratrol-induced apoptosis and cell growth suppression was investigated using uterine sarcoma cells. Resveratrol was found to suppress cell growth in a cancer cell line and to increase the number of apoptotic cells in a dose-dependent manner. Furthermore, the data revealed a dose-dependent downregulation of β-catenin and c-myc, indicating that resveratrol might be a viable therapy option for uterine sarcoma [123].

### 3.18. Lymphoma

The tumor activity of resveratrol on NK/T cell lymphoma cells has also been investigated. It was found that resveratrol suppressed NK/T cell lymphoma cell growth in a dose- and time-dependent manner, and the cell cycle was halted at the S phase [124]. Resveratrol displays diverse mechanisms of action and apoptosis-linked targets in many models. To measure whether resveratrol induces the apoptotic process in Ramos cells, immunoblotting assay was demonstrated against some significant apoptotic markers, precisely, fragmented PARP proteins and active-caspase-3 after different resveratrol treatment. Resveratrol activated a significant increase in cleaved-PARP and active-caspase-3 using 70 μM and 100 μM. Moreover, Ramos cells treated with resveratrol for different time periods showed an important increase in cleaved-PARP and active-caspase-3. Interestingly, resveratrol encouraged a noteworthy increase in the mRNA levels of PUMA and NOXA. These outcomes show that resveratrol can activate caspase-3, encouraging the fragmentation of its downstream target and enhancing the expression of a subset of genes distinguish to be connected to apoptotic events [125]. According to the findings of another investigation, resveratrol was found to activate caspase-3, implying that resveratrol administration can trigger both apoptosis and autophagy.

Resveratrol administration has been found to enhance ROS production, but antioxidants are reported to reduce resveratrol-induced caspase-3 activity and acidic vacuole formation [126]. Resveratrol also causes cell death in a dose-dependent manner, with resveratrol administration causing S-phase cell cycle arrest at lower concentrations. However, at higher concentrations, apoptosis with caspase-3 activation has been detected [173]. Another lymphoma research study found that resveratrol causes caspase-dependent apoptosis by stopping cell-cycle progression in the G_1_ phase and resveratrol-induced cell-cycle arrest and apoptosis that is independent of EBV status [127].

Treatment by resveratrol of human LY8 follicular lymphoma cells causes an accumulation of LY8 cell in apoptosis and the G_0_/G_1_ phase. The expression of BCL6 protein was decreased by resveratrol, concomitant with the increased expression of several BCL6 regulated gene products as well as CD69, p27, and p53. Furthermore, resveratrol reduces expression of Myc in LY8 cells. These outcomes establish that resveratrol inhibits a BCL6-linked pathway and propose that loss of BCL6, a transcriptional repressor frequently translocated in lymphoma expression, may signify a main event primary to the proliferative activities of resveratrol [174]. Resveratrol reduced ROS, inhibited protein synthesis, and inhibited Epstein Barr Virus-induced activation of the redox-sensitive transcription factors such as nuclear factor -kB and AP-1 [128].

### 3.19. Myeloma

The effect of resveratrol on the proliferation, migration, and invasion of multiple myeloma cells was investigated in a special report. The findings showed that the lncRNA NEAT1 was highly expressed in multiple myeloma cells and that resveratrol could effectively inhibit it. Moreover, the overexpression of NEAT1 also caused multiple myeloma cells to proliferate, migrate, and invade; however, resveratrol suppressed this effect [129]. On the MM MM1.S cell line, the combination treatment of resveratrol and rapamycin was investigated. The findings show that rapamycin and resveratrol combination treatment effectively suppressed cell viability when compared to resveratrol or rapamycin monotherapy [175].

In a range of multiple myeloma cell lines, resveratrol produced proliferative action that was dosage and time dependent. A low amount of the proteasome inhibitor carfilzomib (CFZ) was shown to be synergistic with a low dose of resveratrol to cause apoptosis in myeloma cells. Furthermore, resveratrol increased ROS when combined with CFZ. The findings provide a significant foundation for clinics to explore using an autophagy inhibitor in combination treatment for multiple myeloma patients [130]. The antitumor effect of resveratrol against multiple myeloma cell lines was investigated, and the mechanisms involved were also explored. Multiple myeloma cells were suppressed in a dose- and time-dependent manner by this stilbenoid. The expression of the proteins participating in the pathway of apoptotic demonstrated that resveratrol decreased the expression of the antiapoptotic proteins Bcl-xL, Bcl-2, and XIAP and induced the expression of the Bax proapoptotic protein. In addition, resveratrol reduced the levels of XIAP, Bcl-2, and Bcl-xL, proteins in dose-dependent fashion, and complete decline can be observed at resveratrol treatment with 100 µmol/L. Similarly, resveratrol enhanced the Bax protein level in a dose-dependent way. The Bax expression was enhanced at 12.5 µmol/L treatment, and the result was even more obvious at 50 µmol/L [131]. Another research study looked into resveratrol’s effect in multiple myeloma. Resveratrol was shown to have a key role in inhibiting AKT’s constitutive activation. These actions of resveratrol are mediated via inhibition of IB-kinase and phosphorylation of Ikappa B alpha and p65, which suppresses constitutively active NF-B [176].

### 3.20. Melanoma

The significance of resveratrol in melanoma and its interaction with miRNAs were investigated in a study. The findings of the in vitro experiment showed that resveratrol significantly reduced miR-221 expression, which was determined by the overexpression of miR-221 with or without resveratrol therapy. Furthermore, resveratrol’s suppression of miR-221 was demonstrated to be accomplished through modulating NF-B activity. Finally, tumor inhibitory effects of resveratrol were described utilizing an in vivo melanoma model [177].

The effects of resveratrol on melanoma cell viability and melanin production were studied. Treatment of cancer cells with several doses of resveratrol resulted in a concentration-dependent decrease of growth, according to the findings based on the MTT test. Furthermore, resveratrol reduced the expression of the phospho-extracellular signal-related kinase significantly. Altogether, the data show that resveratrol suppresses melanoma cell growth while promoting melanogenesis [178]. After resveratrol therapy, mitogen-activated protein kinases signaling pathways were greatly increased, while mitogen-activated protein kinase inhibitors drastically lowered Cx43 protein expression.

Furthermore, the findings suggest that resveratrol administration in tumors increases Cx43 gap junction communication and enhances the therapeutic benefits of both resveratrol and cisplatin [132]. Another study using melanoma cells found that resveratrol inhibits the survival and migration of melanoma cells via blocking the AKT/mTOR pathway, resulting in autophagy activation. Moreover, resveratrol has been identified as a potential pioneering medication for the treatment of melanoma [133]. Human melanoma cell growth was suppressed by this stilbenoid, which caused cell cycle arrest. Furthermore, resveratrol caused apoptosis in human melanoma cells by upregulating the expression of Bcl-2-associated X protein, according to Western blot study [179]. The inhibitory effects of resveratrol on melanoma cells were investigated using cell viability, apoptosis, and cell morphology. The findings showed that resveratrol effectively suppressed melanoma cell growth by lowering cell viability, inducing apoptosis, and stopping the cell cycle. Ultimately, resveratrol was concluded as inhibiting cancer cell proliferation and was shown to induce apoptosis using induction of cell cycle proteins. However, the duration of treatment was found to be more important than concentration for these effects [134].

### 3.21. Leukemia

The role of resveratrol in a human myelogenous leukemia cell line and its putative molecular pathways was studied. The apoptosis induction was examined to evaluate the inhibitory effect of resveratrol subsequent to inhibition of SphK. After K562 cells were treated with resveratrol by the dose of 20 μM and 40 μM, the apoptotic morphologic changes were noticed when compared with the control. Resveratrol-treated cells showed obvious apoptotic features including membrane integrity loss or deformation, cell shrinkage, nuclear fragmentation, and chromatin compaction of appearance of late apoptosis. The induction of apoptosis of resveratrol in cancer cells was then analyzed, and the results revealed a significant increase of apoptotic cells after exposure to resveratrol. Moreover, in groups of concentrations of 20 μM and 40 μM of resveratrol treatment, the apoptotic cells percentage was 33.17% and 58.28%, respectively, in cancer cells [135].

Another study found that resveratrol might control autophagy in human lymphoblastic leukemia and human promyelocytic leukemia cells, as evidenced by higher levels of the proteins LC3-II and p62/SQSTM1. Furthermore, resveratrol was found to cause apoptosis in examined cell lines, which was linked to mitochondrial membrane potential disturbance, caspase-3 activation, chromatin condensation, and cell nuclei disintegration. Some studies maintain that resveratrol can act as an autophagy modulator and an apoptosis inducer in human leukemia cells [136]. The effect of resveratrol on leukemia cell proliferation and apoptosis was investigated. This compound meaningfully inhibited proliferation activity and increased the activity of caspase-3. Moreover, resveratrol treatment upregulated the expression of PTEN and reduced the expression of the p-AKT protein [137]. The effects of resveratrol plus imatinib mesylate therapy on human chronic myelogenous leukemia cell growth inhibition and apoptosis were investigated.

The results reveal that treatment with resveratrol and imatinib mesylate inhibited K562 cell growth more significantly and resulted in a higher rate of cancer cell apoptosis than treatment with imatinib mesylate alone in the control group. Overall, the findings showed that imatinib mesylate and resveratrol are effective pharmacological therapies for human chronic myelogenous leukemia, suggesting a promising method of preventing cell proliferation and death [180] and that resveratrol has potential as a unique treatment for some forms of chronic and acute leukemia [181].

### 3.22. Osteosarcoma

The effect of resveratrol on osteosarcoma stem cells and putative molecular processes was investigated. Resveratrol was found to play a role in the reduction of osteosarcoma cell viability and carcinogenesis. Furthermore, resveratrol reduced JAK2/Signal transducer and activator of transcription 3 signalings and lowered cytokine production. Again, according to specific data, resveratrol decreased osteosarcoma cell proliferation and tumorigenesis that was associated with cytokine inhibition linked to JAK2/Signal transducer and activator of transcription 3 signaling blocking [59].

Resveratrol inhibits cell migration, proliferation, and invasion and triggers apoptotic cell death in osteosarcoma cells. Moreover, resveratrol possibly downregulates Akt intracellular signaling transduction and nuclear factor κB (NF-κB). Furthermore, the combination of resveratrol and pyrrolidine dithiocarbamate caused synergistic growth inhibition of osteosarcoma [182].

Resveratrol noticeably downregulated the expression of β catenin and meaningfully inhibited cell proliferation. The results of this study established that resveratrol suppressed cancer cells [138]. The role of resveratrol in regulating osteosarcoma cell growth and invasion under hypoxic conditions was investigated. Resveratrol suppresses hypoxia-enhanced proliferation and invasion in osteosarcoma cells by downregulating the HIF-1 protein, according to the findings [139]. According to another study, resveratrol decreases proliferation, while glycolysis triggers apoptosis and reduces osteosarcoma cell invasiveness. Furthermore, as the E-cadherin level increased after treatment with this drug, the expression of related Wnt/catenin signaling pathway target genes was downregulated [183].

The effect of resveratrol on an osteosarcoma cell line was studied, as well as the mechanism behind it. Resveratrol triggered apoptosis in osteosarcoma cells, and both examined cells had lower levels of miR-139-5p than osteoblast cells. Resveratrol therapy can also be used to induce the expression of miR-139-5pin cancer cells [184]. The effects of resveratrol on osteosarcoma cell line proliferation and apoptosis were studied. Resveratrol was found to promote apoptosis in osteosarcoma cells in a dose-dependent manner. Furthermore, the proapoptotic action of resveratrol may be enhanced by a l-asparaginase-induced dietary limitation. Increasing concentrations (0–100 μM) of resveratrol were added to osteosarcoma cell lines, including U-2 OS, HOS, MG-63, Saos-2, and normal osteoblast NHOst cells. Results revealed that at both tested time points, day 3 and day 7, cell growth of all four osteosarcoma cell lines was inhibited by resveratrol in dose dependently, and for the normal osteoblast cells, an apoptotic effect was observed at a 100 μM concentration. This finding explained that resveratrol, at all tested concentrations, caused significant apoptosis on day 3 in all used osteosarcoma cell lines, whereas it produced only a moderate effect on normal osteoblasts [140].

### 3.23. Endocrine Gland-Related Cancer

Anaplastic thyroid carcinoma is a highly deadly, undifferentiated disease that lacks effective treatments. Thyroid malignancies have been treated with retinoic acid to enhance redifferentiation by boosting I131 absorption and radio sensitivity. The therapeutic value of resveratrol in the management of thyroid cancer, either alone or in combination with retinoic acid, was demonstrated in a critical study based on anaplastic thyroid cancer cells. Resveratrol’s ability to overcome retinoic acid resistance in anaplastic thyroid cancer cells was also demonstrated [185]. Resveratrol-treated THJ-16T cells, rather than THJ-11T cells, demonstrated dramatic growth arrest and wide-ranging apoptosis, which was accompanied by increased ROS formation and lower levels of antioxidant enzymes, including SOD and CAT. Furthermore, resveratrol’s potential to boost ROS production and oxidative-associated cellular damages in resveratrol-sensitive THJ-16T cells was discovered [186].

The effects of resveratrol on cell proliferation in thyroid cancer and HFFF2 were examined. Thyroid cell proliferation was meaningfully inhibited by resveratrol treatment with concentrations of 10 and 50 μg/mL. Resveratrol showed a reduction of cellular growth in thyroid cells by 12% when cells were treated with resveratrol with 10 and 50 μg/mL. Moreover, in the comparison of the cancer cells, a human nonmalignant fibroblast cell was used as a control. Resveratrol did not show any significant cellular toxicity in the HFFF2 cell [141]. Resveratrol administration at concentrations ranging from 10 to 50 mol/L inhibited thyroid cancer cell growth in both cell lines in a dose-dependent manner, causing S-phase cell-cycle arrest and apoptosis. Furthermore, resveratrol administration increased the expression of thyroid-specific genes and the NaI symporter in both anaplastic thyroid cancer cell lines. This finding points to resveratrol’s significance in redifferentiation of anaplastic thyroid carcinoma, suggesting that activating Notch1 signaling could be a treatment option for patients with anaplastic thyroid carcinoma. [142].

### 3.24. Brain Cancer

Resveratrol suppressed epithelial-mesenchymal transition and epithelial-mesenchymal transition-linked migratory and invasive ability through Smad-dependent signaling in glioblastoma cells. Moreover, resveratrol clearly inhibited epithelial-mesenchymal transition-induced self-renewal ability of glioma stem cells and inhibited EMT-induced cancer stem cell markers Sox2 and Bmi. Moreover, the inhibitory effect of resveratrol on epithelial-mesenchymal transition in xenograft experiments in vivo was also reported [187]. Resveratrol improved glioblastoma-initiating cells to temozolomide-induced apoptosis and encouraged glioblastoma-initiating cell differentiation [143].

Resveratrol also inhibited epithelial-mesenchymal transition and epithelial-mesenchymal transition-related migratory and invasive capabilities. Furthermore, resveratrol was found to suppress glioma stem cells’ epithelial-mesenchymal transition-induced self-renewal ability as well as epithelial-mesenchymal growth-induced cancer stem cell markers [144]. The effect of resveratrol and paclitaxel combined therapy on TRPM2 activation in glioblastoma cells was investigated in a study. The findings show that indicators for mitochondrial membrane depolarization, apoptosis, mitochondrial function, intracellular steady-state ROS levels, and caspase-3 activity all rose considerably in DBTRG cells after treatment with paclitaxel and resveratrol, respectively. Such medicines, on the other hand, reduced cell viability [188].

### 3.25. Retinoblastoma

The molecular mechanisms by which resveratrol promoted the death of retinoblastoma tumor cells were investigated. The findings show that resveratrol reduced tumor cell viability in a dose- and time-dependent manner, inhibited tumor cell proliferation by causing S-phase growth arrest, and caused apoptotic cell death [145]. It was evidenced that resveratrol treatment of retinoblastoma cells causes dose- and time-dependent reduction in the hyperphosphorylated form of retinoblastoma [189].

## 4. Safety and Efficacy of Resveratrol: Evaluation Based on Human Clinical Trial Study

The role of resveratrol in cancer control has been validated in human clinical trials. Clinical investigations have shown that resveratrol has a therapeutic role in the treatment of cancer. New therapies are being discovered as therapeutic choices for men with biochemically recurrent prostate cancer who wish to defer androgen deprivation therapy. In the phase I portion of a phase I/II study, nonmetastatic biochemically recurrent prostate cancer (BRPC) patients were given increasing doses of MPX (pulverized muscadine grape skin rich in ellagic acid, quercetin, and resveratrol) orally for 28 days, with a follow-up of more than 2 years in cohorts of two patients, with six patients at the highest dose [190]. Initial dose choice was based on preclinical data showed the equivalent of 500 to 4000 mg of MPX to be safe in mouse models. Results revealed that the cohort (*n* = 14, 71% Caucasian, 29% Black) had a median follow-up of 19.2 months, median age of 61 years, and a median Gleason score of 7. Four patients had probably related gastrointestinal symptoms. No other related adverse complication was observed, and one patient showed improvement of chronic constipation. A total of 6 of 14 patients came off the study for disease progression, including 5 metastatic and 1 rising PSA after exposure for a median of 15 months. One patient came off for myasthenia gravis that was unrelated to treatment. Seven patients continue on the study. The lack of dose-limiting toxicities led to the selection of 4 g/d as the highest dose for further study. The lack of statistically significant within-patient change in PSADT and shortening in the PSADT in 36% of the patients raised apprehensions about the efficiency of MPX. The median within-patient PSADT increased by 5.3 months (insignificant). No patients experienced a maintained decline in serum prostate-specific antigen from the baseline. These findings show that 4000 mg of MPX is safe, and an investigative review of a lengthening in PSADT of a median of 5.3 months supports further exploration of MPX [190].

Micronized resveratrol was given as 5g/day for 14 days to patients with colorectal cancer and hepatic metastases who were scheduled for hepatectomy in a pilot study of SRT501. The study’s goal was to determine the formulation’s safety and pharmacodynamics. SRT501 was also shown to be well tolerated. Furthermore, resveratrol (up to 2287 ng/g) was detected in hepatic tissue after SRT501 treatment. In malignant hepatic tissue treated with SRT501, cleaved caspase-3 increased by 39 percent as compared to tissue from placebo-treated patients [191].

A pioneering study was performed to measure the concentrations of resveratrol and its metabolites in the colorectal tissue of humans who took resveratrol. A total of 20 confirmed patients of colorectal cancer consumed 8 daily doses of resveratrol at 0.5–1 g before surgical resection, and resveratrol was noticed to be well tolerated. Normal and malignant biopsy tissue samples were obtained before dosing. Moreover, the parent compound and its metabolites were recognized in colorectal resection tissue. Resveratrol and resveratrol-3-O-glucuronide were recovered from tissues at maximal mean concentrations of 674 and 86.0 nmol/g, respectively. It was noticed that ingesting resveratrol reduced tumor cell proliferation by 5%. The results suggest that daily doses of resveratrol at 0.5 or 1.0 g produce levels in the human gastrointestinal tract of an order of magnitude sufficient to cause anticarcinogenic effects [192]. Trans-resveratrol was studied in people to see if it had a dose-related effect on DNA methylation and prostaglandin expression. A total of 39 adult women with a higher risk of breast cancer were randomized to either placebo or 5–50 mg trans-resveratrol twice daily for 12 weeks in a double-blind study. The methylation status of four cancer-related genes was investigated. The glucuronide metabolite of resveratrol was the most abundant resveratrol species in serum. This research sheds new light on the impact of trans-resveratrol on the breasts of women with a higher risk of breast cancer, including a decrease in the methylation of the tumor suppressor gene RASSF-1α [193].

## 5. Synergistic Effect of Resveratrol in Combination with Anticancer Drugs in Various Types of Cancer

5-fluorouracil, cisplatin, doxorubicin, and paclitaxel are some of the most often utilized cancer medications. Anticancer medications have been reported to induce a variety of side effects, including anemia, hair loss, diarrhea, appetite loss, lethargy, and various physiological and biochemical changes. In this regard, a combination therapy having resveratrol with anticancer drugs may improve its effectiveness, while lowering the toxicity and side effects associated with anticancer drugs (Table 2). Resveratrol has antioxidant potential, decreases cellular oxidative stress levels in a dose-dependent manner, and induces DNA damage. Therefore, it is recommended as a potential therapeutic approach against cancerous cells [194]. The combination therapy or resveratrol given in combination with an anticancer drug shows good results, possibly due to its antioxidant potential.

Human leukemia cells decreased after being exposed to roscovitine, a cyclin-dependent kinase inhibitor. Furthermore, roscovitine therapy was found to reduce the number of hypoploid cells, indicating that cells had undergone apoptosis. In addition, the effects of resveratrol alone and in conjunction with roscovitine were investigated. Surprisingly, synergistic effects were seen after combining treatments and adding resveratrol to the mix after incubation. This combination therapy resulted in a considerable decrease in the frequency of S- and G2/M-phase cells while concurrently increasing the number of G_1_ cells [195]. In malignant mesothelioma cells and normal mesothelial cells, the efficacy of a combination of phytochemicals and clofarabine, an anticancer medication, was investigated. The results of the study demonstrated that combined treatment of resveratrol and clofarabine showed a synergistic antiproliferative effect in malignant mesothelioma cells but not in normal mesothelial cells.

Overall, the results show that the synergistic antiproliferative action of resveratrol and clofarabine is linked to the suppression of Akt and Sp1 activities, suggesting that such a combination might be effective in the treatment of malignant mesothelioma [196]. Another significant study looked at the synergistic effects of resveratrol on cancer. Based on the combination index values, the synergistic combination of doxorubicin and resveratrol was chosen. The resveratrol and doxorubicin combination treatment was found to inhibit the inflammatory response, redox regulation, autophagic flux, and induce apoptosis in breast cancer cells. Additionally, combined dosages of doxorubicin (5 mg/kg b.w.) and resveratrol (10 mg/kg b.w.) inhibited tumor volume with increased life span in Ehrlich ascitic carcinoma cells in mice [197]. Another study found that resveratrol had anticancer properties and that it inhibited cellular survival by downregulating canonical Wnt signalling proteins in treated breast cancer cells.

Furthermore, cotreatment with salinomycin significantly enhanced resveratrol’s anticancer properties. In addition, cells treated with a combination of resveratrol and salinomycin showed significant downregulation of canonical Wnt signaling proteins and vimentin which is a hallmark of epithelial-mesenchymal transition [113]. In vitro, resveratrol greatly increased the anticancer efficacy of arsenic trioxidein. Furthermore, an isobolographic study revealed a synergistic effect between arsenic trioxide and resveratrol. In the combined therapy group, there was more apoptosis and ROS production and identical synergistic effects were found in mice in vivo [198].

In another study, researchers looked at how resveratrol interacts with docetaxel and doxorubicin, as well as the molecular basis of this interaction in solid tumor cell lines. The findings show that resveratrol, when combined with doxorubicin and docetaxel, significantly enhanced the efficacy of both chemotherapeutic drugs. The combination of resveratrol with doxorubicin or docetaxel increased the expression of Bax and Bcl-2 in all tested cell lines according to real-time PCR results [199]. The tumor impact of resveratrol and 5-fluorouracil (5-FU) in the treatment of liver cancer was explored. Resveratrol suppresses the development of murine hepatoma cells and induces H_22_ cells to arrest in the S phase. Furthermore, when 5-FU was administered in conjunction with resveratrol, it inhibited tumor development more effectively in hepatoma-bearing mice [200]. The anticancer potential of the combination of 5-fluorouracil and resveratrol was examined. The mechanisms of action were also investigated. The combination of resveratrol and 5-FU was found to have synergistic activity, resulting in tumor regression. Furthermore, resveratrol enhanced the growth inhibitory effect of 5-FU on cancer cells when investigated using in vitro. In addition, the tumor regression rate in the combination group increased significantly following four weeks of therapy [201]. Another experiment looked at whether resveratrol may boost the anticancer effects of melphalan. In human breast cancer cells, resveratrol was reported to enhance the cytotoxic effects of melphalan. This was linked to resveratrol’s capacity to make MCF-7 cells more susceptible to melphalan-induced apoptosis [202].

## 6. Strategies to Overcome the Low Bioavailability of Resveratrol

Despite its efficacy and safety, the function of resveratrol in the treatment and prevention of illnesses is still debatable due to its low bioavailability and quick metabolism. Around 75% of resveratrol is absorbed in humans after oral dosing, most likely by transepithelial diffusion. However, due to rapid and extensive metabolism in the gut and liver, oral bioavailability is reduced to less than 1% [203,204]. The absorption, bioavailability, and metabolism of 14C-resveratrol following oral and intravenous dosages were investigated. However, only minor levels of unaltered resveratrol less than 5 ng per ml were detected in plasma, with the majority of the oral dosage recovered in urine [205]. As a result, multiple studies have demonstrated that there are effective ways to overcome the challenges of poor absorption, quick metabolism, and rapid systemic clearance.

Enhancing the rate of resveratrol absorption into enterocytes and lowering intracellular metabolism are two popular strategies for increasing bioavailability from oral resveratrol administration [206,207,208,209]. Several studies show that different resveratrol formulations, such as encapsulation, micelles, liposomes, and nanoparticles, increase resveratrol bioavailability. Furthermore, nanonization of drug particles to produce nanocrystals is a promising strategy for improving solubility, physical and chemical stability, compatibility with oral dosage forms, and oral bioavailability [210].

In colon cancer cells, the antiproliferative effects of free resveratrol and resveratrol-loaded lipid-core-nanocapsule were investigated. The study’s findings clearly showed that cytotoxicity is dosage and time dependent. Furthermore, resveratrol has been shown to cause cancer cell death via the apoptotic pathway. Furthermore, resveratrol was shown to have a significant apoptotic impact. Free resveratrol produced almost 15% cell death, but resveratrol-loaded lipid-core-nanocapsule generated a significant 36% cell apoptosis, indicating that it has a greater anticancer impact in cancer cells [211].

Another study used liposomes to coencapsulate pristine resveratrol with cyclodextrin-resveratrol enclosure complexes in the lipophilic and hydrophilic compartments to develop and optimize a new drug carrier. The findings showed that new nano-formulations had 100% drug release in comparison with free resveratrol and traditional liposomal formulations with a drug release profile of 40–60%. The cytotoxicity of resveratrol encapsulated liposomes in colon cancer cell lines was also tested in vitro. Compared to free resveratrol, the cytotoxicity profile of liposomes was shown to be dosage dependent and was reported to be improved [212].

In vitro, trans-resveratrol-loaded lipid-core nanocapsules were also investigated and found to decrease the viability of glioma cells to a higher amount than resveratrol. The vitality of glioma cells in vitro was considerably decreased by trans-resveratrol-loaded lipid-core nanocapsules. The administration of nanocapsules was not found to be lethal to hippocampus organotypic cultures, which are a model of healthy brain cells, indicating that its selectivity for using it for cancer cells. Nanocapsules reduced the viability of glioma cells by inducing apoptotic cell death. Overall, the findings suggest that resveratrol nano-encapsulation increases its glioma action [213].

## 7. Conclusions

Cancer is a disease that is affected by a number of factors and is among the major causes of a high and continually rising death rate across the world. The currently used mode of treatment such as chemotherapy, radiation, and surgical removal of tumors shows serious side effects. In this regard, resveratrol boasts an extensive spectrum of cancer preventive effects through modulating cell cycle, autophagy, apoptosis, angiogenesis, and other cell signaling pathways. Moreover, the antioxidant potentiality of resveratrol plays a significant role in the inhibition of free radicals, reduces the oxidative stress and prevents the cancer development and progression. Despite its efficacy and safety, resveratrol’s significance in disease treatment and prevention is still debatable due to its low bioavailability and rapid metabolism. Encapsulation, liposomes, micelles, and nanoparticles are some of the strategies have been used to enhance the bioavailability of resveratrol.

To date, few clinical studies on resveratrol in human cancer therapy are available. More studies based on clinical trials should be warranted to explore the role of resveratrol in cancer management and its exact mechanism of action in cancer prevention.

## Figures and Tables

**Table 1:** The role of resveratrol in numerous cancers through modulating various cell signaling pathways.

Cancers	Finding/Outcome of the Study	Ref.
Head and neck cancer	Resveratrol enhanced the efficiency of cisplatin and irradiation and resultant decrease of cancer progression	[68]
Oral cancer	Adhesion of cancer cells treated with resveratrol was decreased and invasive abilities of cancer cells treated with resveratrol were decreased	[71]
	Resveratrol inhibited cancer through induction of apoptosis	[73]
Oesophagus cancer	Resveratrol inhibited cancer cell growth in a dose-dependent way through prompting cell cycle arrest	[75]
	Treatment of cancer cells with resveratrol the PRs of Bcl-2 proteins were seemingly reduced	[77]
Lung cancer	Apoptosis was induced in TRAIL-resistant lung cancer cells with a cotreatment of resveratrol	[78]
	Resveratrol caused the tumor outcome via decreasing cell proliferation and promoting cell apoptosis	[79]
	Resveratrol concentration and time dependently reduced cancer cell viability	[80]
	Resveratrol synergistically increased the tumor effects of erlotinib	[81]
Gastric cancer	Resveratrol inhibited the interleukin-6 induced cancer cell invasion	[82]
	Resveratrol inhibited the growth of cancer cells through preventing the Wnt signaling pathway	[83]
	Resveratrol was able to significantly inhibit the viability of cancer cells	[84]
	Resveratrol-induced inhibition of cancer SNU-1 cell proliferation	[85]
Gall bladder cancer	Resveratrol clearly decreased the proliferation in concentration as well as time-dependent manner and resveratrol induced apoptosis of tumor cells	[86]
Bile duct cancer	IL-6 indeed promoted the cell migration of invasive cancer cells; the resveratrol powerfully neutralized this effect both in cancer cells	[87]
Liver cancer	Resveratrol played a role in the inhibition of the proliferation and mobility of carcinoma cells via prompting autophagy	[88]
	Prevention of cancer cell migration, tumor suppressor gene DLC1 Rho GTPase activating protein level was enhanced with resveratrol treatment	[56]
	Resveratrol importantly controlled tumor growth	[89]
	Resveratrol established a potential protective effect on cancer cells in a lipid overload state	[90]
Pancreas cancer	Resveratrol- and apocynin-treated hamsters exhibited important decrease in the incidence of cancer	[91]
	Resveratrol inhibited the cell proliferative ability in a dose- and time-dependent manner.	[92]
	Resveratrol treatment induced apoptosis, inhibited tumor growth, and increased the Bax expression	[93]
	Resveratrol derivative played role in the induction of dose-dependent apoptosis in cancer cell lines	[94]
Colon cancer	Resveratrol induced cytotoxicity on cancer cells	[95]
	Resveratrol efficiently inhibited cell proliferation and promoted cell apoptosis	[96]
	Resveratrol caused inhibition of cell proliferation interrelated with an induction of apoptosis	[97]
	Cancer cells exposed to resveratrol displayed meaningfully lower cyclooxygenase-2 and prostaglandin receptor expression	[98]
	PPARγ playe a role in resveratrol-induced apoptosis	[99]
Renal cell carcinoma	Sitagliptin/resveratrol combination might signify a useful therapeutic modality for improvement of clear cell renal cell carcinoma	[100]
	Resveratrol suppressed renal cell carcinoma and migration and promoted carcinoma apoptosis	[101]
	resveratrol suppressed renal cell carcinoma migration, cell proliferation, and invasion	[102]
	Resveratrol induced S-phase cell-cycle arrest and caused induction of apoptosis	[103]
Prostate cancer	Resveratrol and its combination with bicalutamide significantly reduced cell viability	[104]
	Resveratrol was able to downregulate the levels of the endogenously expressed ARV7 and androgen receptor target gene mRNAs in prostate cancer cells	[105]
	Resveratrol, DTX, and a combined drug treatment upregulated the proapoptotic genes	[64]
Bladder cancer	Resveratrol was revealed to significantly inhibit the expression and secretion of matrix metalloproteinase-2	[106]
	Resveratrol decreased cell proliferation and induced DNA damage	[107]
	The effect of resveratrol on cancer cell apoptosis was due to miR-21 regulation of the Akt/Bcl-2 signaling pathway	[108]
	Resveratrol treatment decreased the expression of thevascular endothelial growth factor	[109]
Breast cancer	Resveratrol-induced chemosensitivity, cell cycle, and apoptosis were arrested	[110]
	Proanthocyanidins and resveratrol synergistically inhibited breast cancer cells via inducing apoptosis and modulating DNA methylation	[111]
	Suppression of EZH2 expression through ERK1/2 dephosphorylation was significant for the antiproliferative activities of resveratrol against breast cancer cells	[112]
	Cell cycle arrest, caspase activation as well apoptotic induction in cells treated with resveratrol-salinomycin combination established the efficiency of the combination	[113]
Endometrial cancer	Resveratrol treatment inhibited the growth of cancer cells in a dose-dependent manner	[114]
	Resveratrol arbitrated suppression of a functional activity of progesterone receptor as established by downregulation of alpha one integrin expression	[115]
Cervix cancer	Resveratrol treatment with various concentrations caused increased cell cycle arrest	[116]
	Resveratrol showed a role in the inhibition of both NF-κB and AP-1-mediated metalloproteinase-9 expression	[117]
	Long treatment of resveratrol induced cytosolic translocation of cytochrome c, caspase-3 activation, and apoptotic cell death	[118]
	Resveratrol pretreatment caused inhibition of cell division and induced an early S-phase cell-cycle checkpoint arrest	[119]
Ovarian cancer	ARHI was expressed in low levels in ovarian cancer cell lines, which was enhanced after resveratrol treatment accompanied by growth arrest	[120]
	Resveratrol analogues decreased the expression of epithelial mesenchymal transition markers	[121]
	Resveratrol induced apoptotic cell death in dose- and time-dependent manners	[122]
Uterine cervix	Resveratrol inhibited cell proliferation in the cancer cell line, and the number of apoptotic cells increased in a resveratrol dose-dependent manner	[123]
Lymphoma	Resveratrol suppressed the phosphorylation level of AKT and Stat3	[124]
	Resveratrol played a role as proliferative and proapoptotic activity	[125]
	Resveratrol treatment increased reactive oxygen species generation, and the reactive oxygen species scavenger could decrease both the resveratrol-induced caspase-3 activity and the formation of acidic vacuoles	[126]
	Resveratrol induced caspase-dependent apoptosis via arresting cell-cycle progression	[127]
	Resveratrol played a role in the inhibition of protein synthesis, decreasing reactive oxygen species levels	[128]
Myeloma	NEAT1 overexpression induced proliferation, migration, and invasion of multiple myeloma cells, although resveratrol neutralized its effect	[129]
	Resveratrol caused proliferative activity in a dose- and time-dependent manner	[130]
	Resveratrol inhibited proliferation of myeloma cells in a dose- and time-dependent manner	[131]
Melanoma	Resveratrol treatment inhibited proliferation and promoted melanogenesis of melanoma cells	[164]
	Treatment of resveratrol in a tumor caused an increase in Cx43 gap junction communication and improved the combination of resveratrol and cisplatin therapeutic effects	[132]
	Resveratrol may assist as a pioneering therapeutic for melanoma treatment	[133]
	Resveratrol inhibited cancer cell proliferation and triggered apoptosis	[134]
Leukemia	Resveratrol inhibited the proliferation as well as induced apoptosis	[135]
	Resveratroled act as an autophagy modulator and an apoptosis inducer in human leukemia cells	[136]
	Resveratrol treatment upregulated the expression of PTEN and reduced the expression of p-AKT protein	[137]
Osteosarcoma	Resveratrol inhibited cancer cell proliferation and tumorigenesis ability	[59]
	Resveratrol suppressed the cancer cells by preventing the canonical Wnt signaling pathway	[138]
	Resveratrol inhibited the hypoxia-enhanced proliferation, epithelial to mesenchymal transition process, and the invasion in osteosarcoma	[139]
	Pro-poptotic effect of resveratrol might be improved by nutrition restriction elicited by l-asparaginase	[140]
Thyroid cancer	Resveratrol enhanced cell death induced by (131)I on thyroid cancer cell	[141]
	Resveratrol treatment suppressed thyroid carcinoma cell growth in a dose-dependent manner	[142]
Glioblastoma	Resveratrol improved glioblastoma-initiating cells to temozolomide-induced apoptosis	[143]
Glioma	Resveratrol clearly inhibited EMT-induced self-renewal ability of glioma stem cells	[144]
Retinoblastoma	Resveratrol induced a dose- and time-dependent decrease in tumor cell viability and also caused inhibition of proliferation	[145]

**Table 2:** Combination of resveratrol with anticancer drugs potentially enhance the efficacy of anticancer drugs.

Resveratrol + Anticancer Compound/Drugs	Type of Cancer	Outcome of the Study	Ref.
Resveratrol and roscovitine	Leukemia	Synergistic effects were noticed after combined treatment and consecutive postincubation in the presence of resveratrol. Such combination treatment caused significant reduction of the frequency of the S- and G (2)/M-phase cells	[195]
Resveratrol and clofarabine	Mesothelioma	Resveratrol and clofarabine showed synergistic antiproliferative effect in malignant mesothelioma cells	[196]
Doxorubicin and resveratrol	Breast cancer	Combined treatment was also established to inhibit the inflammatory response, redox regulation and caused apoptosis in breast cancer cells	[197]
resveratrol with docetaxel and doxorubicin	Solid tumor	Resveratrol in combination with doxorubicin and docetaxel meaningfully increased powers of both chemotherapeutic agents.	[199]
resveratrol and in combination with 5-FU	Liver cancer	Enhanced inhibition of tumor growth by 5-FU was also observed in hepatoma 22 bearing mice when 5-FU was given in combination with resveratrol	[200]
5-fluorouracil and resveratrol	tumor	Resveratrol and 5-fluorouracil established synergistic efficacy, causing tumor regression. Moreover, there was clear confirmation of resveratrol enhancing the growth inhibitory effect of 5-FU on the cancer cells	[201]
Resveratrol and melphalan	Breast cancer	Resveratrol potentiated the cytotoxic effects of melphalan in human breast cancer cells	[202]

## Data Availability

The data used to support the findings of this study are included within the article.
